# Organized Chronic Subdural Hematoma Mimicking Acute Epidural Hematoma

**DOI:** 10.1155/2023/6645752

**Published:** 2023-11-27

**Authors:** Jun Cao, Zhichun Wang, Cegang Liu, Jun Shen, Jincheng Fang

**Affiliations:** Department of Neurosurgery, The First Affiliated Hospital of Wannan Medical College (Yijishan Hospital of Wannan Medical College), Wuhu 241001, China

## Abstract

**Background:**

Chronic subdural hematoma is a common disease in neurosurgery, but organized chronic subdural hematoma is rarely seen clinically. This article reports a case of misdiagnosis of organized chronic subdural hematoma as acute epidural hematoma. Through literature review, the causes of misdiagnosis and the treatment methods of organized chronic subdural hematoma are discussed. *Case Description*. A 70-year-old male patient was admitted to the hospital due to headache and dizziness after head trauma. Emergency head CT reported “left frontotemporal parietal epidural hematoma.” Because the head CT showed that the hematoma occupying effect was obvious, an emergency “intracranial hematoma evacuation” was performed. After opening the skull during the operation, no epidural hematoma was seen. Upon incision of the dura mater, the outer membrane of organized chronic subdural hematoma was found. When the outer membrane was cut open, a large amount of reddish-brown silt-like materials was found in the capsule cavity. The inner membrane was not forcibly removed. Postoperative head CT showed that the organized chronic subdural hematoma was basically cleared.

**Conclusion:**

The early symptoms of organized chronic subdural hematoma are atypical, with insidious onset and easy misdiagnosis. By carefully inquiring about the medical history and carefully reading the head CT, such misdiagnosis can be avoided. Craniotomy is currently an important treatment option for organized chronic subdural hematoma.

## 1. Introduction

Chronic subdural hematoma (CSDH) often begins to develop more than 3 weeks after trauma. CSDH is generally located between the dura and arachnoid membranes, and its incidence accounts for approximately 10% of intracranial hematoma [1]. Hematomas often occur on the convex surface of the frontal, parietal, and temporal hemispheres and are common in children and the elderly [2]. However, organized chronic subdural hematoma (OCSH) is extremely rare in clinical practice, and there are few literature reports [3]. Due to atypical early symptoms, insidious onset, and slow progression of symptoms in patients with OCSH, this type of patient is easily misdiagnosed. We report a case of OCSH misdiagnosed as acute epidural hematoma and discuss the manifestations and surgical treatment of the disease in combination with relevant literature.

## 2. Case Description

**Figure 1:**
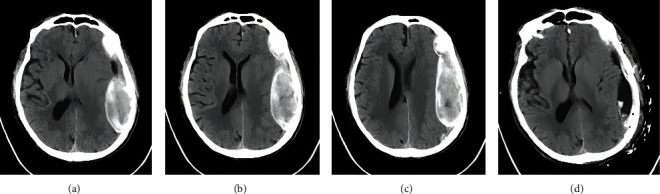
(a)–(c) show the soft tissue windows of the emergency axial head CT without IV contrast of the patient on admission, showing a huge fusiform high-density shadow in the left frontotemporal parietal region, which is very similar to the common CT findings of acute epidural hematoma. (d) shows the soft tissue window of the axial head CT without IV contrast on the first postoperative day, showing that the organized subdural hematoma was almost cleared and the compression of the brain tissue was relieved.

The patient, a 70-year-old Chinese male, was admitted to the emergency department of our hospital due to headache and dizziness for 5 hours after head trauma caused by accidentally falling. Physical examination showed that the consciousness was clear, and the muscle strength of the right limb was grade 4. The skin contusion and swelling on the forehead were found. He had no previous history of special diseases other than hypertension. Emergency head CT reported “left frontotemporal parietal epidural hematoma” (Figure 1).

**Figure 2:**
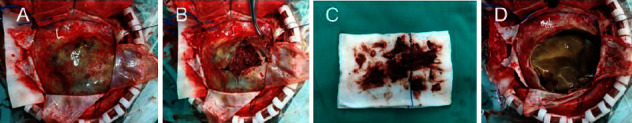
(A) The outer membrane of the organized chronic subdural hematoma was seen after the dura mater was cut open. (B) The outer membrane of the organized chronic subdural hematoma was cut open, and a large amount of reddish-brown silt-like solid blood was seen. (C) Solid hematoma that has been removed. (D) After evacuation of the hematoma, a yellowish inner membrane of the organized hematoma was seen.

Because the head CT showed that the hematoma occupying effect was obvious, an emergency “intracranial hematoma evacuation” was performed under general anesthesia. After opening the skull during the operation, the dura mater was intact, the tension of dura mater was high, and no epidural hematoma was seen. Upon incision of the dura mater, the outer membrane of organized chronic subdural hematoma was found. The outer membrane was tightly adhered to the dura mater, which was light yellow and leather-like in texture. When the outer membrane was cut open, a large amount of reddish-brown silt-like materials were found in the capsule cavity (Figure 2). Part of the outer membrane and contents were taken for pathological examination.

**Figure 3:**
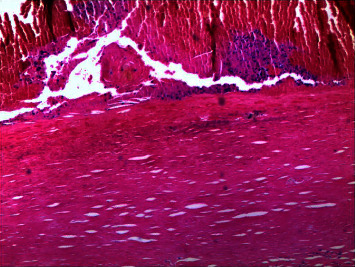
Postoperative pathology showed cystic tissue, fibrous collagen tissue with lymphocyte infiltration, and blood clots in the cyst. Picture was taken at 200 magnification.

The inner membrane rarely adhered to the arachnoid membrane and was easy to separate, but there was much blood oozing during separation. After resection of the outer membrane and removal of organized tissue within the capsule cavity, an attempt was made to separate the inner membrane of the hematoma from the surface of the brain tissue. However, when the inner membrane was separated, there was much blood oozing, and the inner membrane was not forcibly removed. Postoperative head CT showed that the organized chronic subdural hematoma was basically cleared, and the inner membrane was residual. Postoperative pathology showed cystic tissue, fibrous collagen tissue with lymphocyte infiltration, and blood clots in the cyst (Figure 3). The patient recovered well and was discharged from the hospital 2 weeks after surgery. The long-term follow-up CT scan of the patient showed the long-term presence of a partially organized hematoma in the subdural space, containing a small amount of air, which is related to the failure to completely remove the inner membrane of the organized chronic subdural hematoma in this case.

## 3. Discussion

Chronic subdural hematoma generally refers to hematoma between the dura mater and the arachnoid membrane that begins to appear more than 3 weeks after head trauma. Organized chronic subdural hematoma is rare in clinic, and it is more common in the elderly, accounting for about 6.51% of all chronic subdural hematoma [4].

### 3.1. Pathogenesis

The pathogenesis of OCSH remains unclear. Shrestha et al. [5] concluded that ongoing chronic inflammatory process leads to the formation of the membrane of OCSH which consists of neovascularization and tissues like fibroblasts and granulation tissues. The neocapillary blood vessels in the outer membrane of the subdural hematoma formed after trauma continue to rupture and bleed after excessive fibrinolysis. After blood enters the hematoma cavity, hemosiderin and calcium salt are continuously deposited, and the hematoma volume gradually expands, the hematoma cavity becomes a solid substance without tissue structure. Eventually, the fibrous granulation tissue of the membrane was overproliferated and organized, and the membrane gradually thickened. The outer membrane of OCSH adheres closely to the dura mater and is prone to bleeding after dissection; the inner membrane has little adhesion to the arachnoid membrane and is easy to separate. The content of the hematoma is a solid hematoma or organized tissue, presenting as a reddish-brown sediment-like, cheese-like substance with a small amount of yellowish-brown liquid or reddish-brown colloidal substance [6].

### 3.2. Imaging Characteristics

The CT manifestations of organized chronic subdural hematoma are crescent, semilunar, or fusiform low or equal or high-density shadows under the inner plate of the skull, with annular septal or strip-shaped high-density shadows in some parts [7]. The midline structure shifts to the opposite side to varying degrees, the lateral ventricle is compressed and deformed, and most of the inner membrane presents high-density shadows. A fully organized hematoma appears as a uniform low-density shadow on CT imaging, and the cerebrospinal fluid signal between the organized hematoma and the brain tissue could be less dense. It is difficult to differentiate organized chronic subdural hematoma from chronic subdural hematoma solely based on imaging, but for organized chronic subdural hematoma, in head CT without IV contrast, there may be calcification or cord-like separation within the hematoma. Head MRI can further clarify the diagnosis. MRI of the head shows a mixed cord-like separation within the hematoma cavity, which is an important feature of hematoma organization.

### 3.3. Pitfalls

In the emergency management of this case, we misdiagnosed the organizing chronic subdural hematoma as an acute epidural hematoma (EDH). By reviewing and analyzing the characteristics of this case, it was found that there were two things that were easy to cause doctors to misdiagnose. Firstly, the patient had a history of head trauma caused by fall and had an acute onset. Secondly, the location of this organized chronic subdural hematoma is relatively consistent with that of a common epidural hematoma caused by hemorrhage from the middle meningeal artery. Preoperative head CT revealed mixed high-density shadows, indicating that there is still a possibility of active bleeding in the epidural hematoma and that the condition may progress, misleading doctors to emergency craniotomy.

We also found that there were some points of differentiation between this case and general epidural hematoma. First of all, the patient's preoperative state of consciousness did not closely match the head CT images, and there was no clinical manifestation of “intermediate awake period,” which is typical of “coma, awake, recoma” of epidural hematoma. Secondly, acute epidural hematoma is usually caused by severe trauma, and the site of occurrence is mostly the focus of head trauma, so scalp hematoma or skull fracture can often be found in the local area, and the fracture often passes through the dural blood vessels or venous sinus. Acute epidural hematoma is often with sharp boundaries on CT images. Careful reading of the preoperative head CT of this case showed that the hematoma border was slightly rough, which was not consistent with epidural hematoma and did not suggest skull fracture. The key points for differential diagnosis between epidural hematoma and OCSD are listed in Table 1. Therefore, this kind of misdiagnosis can be avoided by carefully asking the medical history, carefully reading the head CT, and considering the patient's consciousness.

In our case, according to intraoperative findings, the inner and outer membranes of the organized chronic subdural hematoma are intact. No obvious fresh blood clots were observed in the capsule during surgery, but rather a large amount of reddish-brown silt-like material without organizational structure. Combined with intraoperative findings, rebleeding into a chronic subdural condition can be excluded.

### 3.4. Surgical Management and Complications

Currently, it is believed that drilling and drainage is not effective for the treatment of OCSH, and craniotomy is currently an important treatment method [8]. The size of the craniotomy skull window should be as large as possible to expose the edge of the hematoma in order to facilitate the removal of the membrane of the organized hematoma.

The failure to perform total membranectomy in this case is related to the failure to fully expose the hematoma boundary during operation. Kayaci et al. [9] report that the main complications of craniotomy and membranectomy include rebleeding and epilepsy. Rebleeding may be due to severe injury during craniotomy and membranectomy, especially due to severe adhesion of the membrane to the dura mater and the tight binding of the membrane to the bridging vein near the midline. It may also be due to long-term compression of local brain tissue, and once the compression is relieved, a similar phenomenon of “cerebral hyperperfusion” will occur, causing acute cerebral vasodilation and widespread small blood vessel rupture and hemorrhage.

The reason why epilepsy is prone to occur after craniotomy is considered to be due to slight damage to the surface of the cerebral cortex during the membranectomy [10]. If the membrane is thick and tough, it should be carefully peeled off under a microscope if possible. If necessary, the membrane can be preserved, and only a radial incision is made on the membrane to facilitate postoperative brain tissue reposition. We should not blindly pursue total resection of the membrane and damage the cerebral cortex.

## 4. Conclusion

We report a rare case of organized chronic subdural hematoma misdiagnosed as acute epidural hematoma. Neurosurgeons should consider the possibility of OCSH, although the incidence of OCSH is low. Early recognition of OCSH and determination of proper treatment may lead to improved survival. Craniotomy is currently an important treatment option for OCSH.

## Figures and Tables

**Table 1:** Contrasting and comparing EDH and OCSH.

	History of trauma	State of consciousness	Clinical manifestation	CT imaging
EDH	Usually caused by severe head trauma	Coma, awake, recoma	Usually have scalp hematoma and skull fracture	Fusiform high-density shadow with sharp boundary
OCSH	Usually caused by minor head trauma	Usually awake	No scalp hematoma and no skull fracture	Curved or crescent low-density or mixed-density shadow with rough boundaries

EDH: epidural hematoma; OCSH: organized chronic subdural hematoma.

## Data Availability

The original contributions presented in the study are included in the article; further inquiries can be directed to the corresponding author.
